# Modeling allele-specific expression at the gene and SNP levels simultaneously by a Bayesian logistic mixed regression model

**DOI:** 10.1186/s12859-019-3141-6

**Published:** 2019-10-28

**Authors:** Jing Xie, Tieming Ji, Marco A. R. Ferreira, Yahan Li, Bhaumik N. Patel, Rocio M. Rivera

**Affiliations:** 1 Department of Statistics, University of Missouri at Columbia, Columbia, 65211 MO USA; 2 Department of Statistics, Virginia Tech, Blacksburg, 24061 VA USA; 3 Division of Animal Science, University of Missouri at Columbia, Columbia, 65211 MO USA

**Keywords:** Allelic imbalance, Hierarchical generalized linear mixed model, High-throughput sequencing experiments, Single nucleotide polymorphism

## Abstract

**Background:**

High-throughput sequencing experiments, which can determine allele origins, have been used to assess genome-wide allele-specific expression. Despite the amount of data generated from high-throughput experiments, statistical methods are often too simplistic to understand the complexity of gene expression. Specifically, existing methods do not test allele-specific expression (ASE) of a gene as a whole and variation in ASE within a gene across exons separately and simultaneously.

**Results:**

We propose a generalized linear mixed model to close these gaps, incorporating variations due to genes, single nucleotide polymorphisms (SNPs), and biological replicates. To improve reliability of statistical inferences, we assign priors on each effect in the model so that information is shared across genes in the entire genome. We utilize Bayesian model selection to test the hypothesis of ASE for each gene and variations across SNPs within a gene. We apply our method to four tissue types in a bovine study to de novo detect ASE genes in the bovine genome, and uncover intriguing predictions of regulatory ASEs across gene exons and across tissue types. We compared our method to competing approaches through simulation studies that mimicked the real datasets. The R package, BLMRM, that implements our proposed algorithm, is publicly available for download at https://github.com/JingXieMIZZOU/BLMRM.

**Conclusions:**

We will show that the proposed method exhibits improved control of the false discovery rate and improved power over existing methods when SNP variation and biological variation are present. Besides, our method also maintains low computational requirements that allows for whole genome analysis.

## Background

In a diploid cell, the two alleles of a gene inherited from maternal and paternal parents express roughly equally for most genes. However, research has uncovered a group of genes in the genome where two copies of a gene express substantially differently, a phenomenon known as allelic imbalance. One such example involves imprinted genes whose allele expression is based on the parent of origin [1, 2]; that is, imprinted genes are mainly or completely expressed from either the maternally or paternally inherited allele but not both, so the total expression from genomic copies is the appropriate amount for healthy and viable organisms [3]. Another prominent example is X-chromosome inactivation in mammals [4, 5], where one copy of the X chromosome is inactivated in female cells to maintain the same dosage of X-linked genes compared to male cells. The choice of which X chromosome is silenced is random initially, but once chosen, the same X chromosome remains inactive in subsequent cell divisions. In a third and rather random case, allelic imbalance occurs when there are mutations in *cis*-regulatory regions of one allele, leading to differential expression of two alleles [6, 7].

Allelic imbalance affects approximately 5-10% of genes in the mammalian genome [5], but it is not biologically clear what series of mechanisms a cell employs to precisely initiate allele-specific expression (ASE) during fetal development and consistently maintain it through a lifetime. Several common congenital human disorders are caused by mutations or deletions within these ASE regions, such as Beckwith-Wiedemann syndrome (BWS) [8, 9], which characterizes an array of congenital overgrowth phenotypes; Angelman syndrome [10], which characterizes nervous system disorders; and Prader-Willi syndrome, in which infants suffer from hyperphagia and obesity.

To understand the molecular mechanisms underlying ASEs and human developmental defects due to misregulated ASE regions, a powerful and accurate computational algorithm to detect genome-wide ASEs is urgently needed. The binomial exact test, employed in AlleleSeq [11], is one of the most widely used methods to test ASEs due to its simplicity. [12] uses analysis of variance (ANOVA) in their proposed pipeline Allim. [13] fits a mixture of folded Skellam distributions to the absolute values of read differences between two alleles. However, these abovementioned statistical methods draw conclusions based on observations produced from one gene; due to the expensive cost of acquiring tissue samples and sequencing experiments, most laboratories can only afford three or four biological replicates. Depending on sequencing depth, genes may also have low read counts, limiting the power of the aforementioned methods.

In searching for more powerful and reliable ASE detection methods, several groups have proposed Bayesian approaches to share information across genes and thus improve gene-related inferences on average. For instance, the MBASED method [14] and the QuASAR method [15] all assume the read counts follow binomial distributions with a beta prior on the probability parameter. In their statistical models, they assume that ASE of a gene or a region is constant across SNPs. However, ASE is known to vary within a gene due to alternative splicing [16, 17], which is essentially universal in human multi-exon genes that comprise 94% of genes overall [17, 18]. Therefore, a highly desirable feature of ASE detection methods is identification of ASE genes and ASE variations within genes across multiple exons. [19] developed a flexible statistical framework that satisfied this requirement. It assumes a binomial distribution with a beta prior. Additionally, it places a two-component mixture prior on the parameters of the beta-binomial model. A Markov chain Monte Carlo (MCMC) method was adopted to compute posterior probabilities for inferences of genes and SNPs. However, due to the extensive computational power required in the MCMC calculation for one gene and the large number of genes in the entire genome, this method is not empirically appealing. Other relevant methods include the EAGLE method [20] that detects associations between environmental variables and ASEs, the WASP method [21] that addresses incorrect genotype calls, and the RASQUAL method [22] that detects gene regulatory effects.

In this paper, we propose a new statistical method that addresses the abovementioned challenges. Specifically, our proposed approach can detect ASE genes and ASE variations within genes simultaneously while maintaining a low computational requirement. Coupled with exon and RNA transcript information, our statistical predictions produce detailed, biologically relevant, intriguing results that enable researchers to examine the molecular mechanisms of ASE regulation in detail.

Particularly, we model the logistic transformation of the probability parameter in the binomial model as a linear combination of the gene effect, single nucleotide polymorphism (SNP) effect, and biological replicate effect. The random SNP effect permits ASE to vary within a gene; the random replicate effect accounts for extra dispersion among biological replicates beyond binomial variation. To overcome the low number of biological replicates and/or low number of read counts of a gene, we propose a hierarchical model with a Gaussian prior on the fixed gene effect and inverse gamma priors, respectively, on the variance components of the random SNP and replicate effects. We test hypotheses via Bayesian model selection method based on model posterior probabilities. To compute posterior probabilities, we propose combining the empirical Bayes method and Laplace approach to approximate integrations, leading to substantially reduced computational power requirements compared to MCMC. We illustrate the utility of our proposed method by applying it to the bovine genome in [23], which motivated our study; findings reveal for the first time highly detailed information regarding the testing results for whole-genome ASEs, unveiling inspiring ASE variations across exons and across tissue types. To compare our method with existing approaches, we simulate data that mimic real datasets to ensure that the comparison results can be reproduced in practice. The proposed method outperforms existing methods in false discovery rate (FDR) control of detecting ASEs and variations therein across SNPs. We call our method the Bayesian Logistic Mixed Regression Model (BLMRM) method. The R package, BLMRM, for the proposed method is publicly available for download at https://github.com/JingXieMIZZOU/BLMRM.

## Results

### Application for the de novo identification of ASE and imprinted genes in bovine

Most of the imprinted genes identified to date have been in the mouse [24]. Original work, identified the non-equivalency of the parental alleles by generating embryos which only had maternal chromosomes (gynogenotes and parthenogenotes) or paternal chromosomes (androgenotes) [25, 26]. By doing this, investigators identified which genes are expressed exclusively from each chromosome. Other studies used mice which had various types of genetic rearrangements including translocations, duplications and deletions and noticed that the direction in which the allele was inherited (either through the mother or the father) mattered for the successful development and wellbeing of the offspring [27]. Subsequent work turned to genetic manipulations to identify the function of imprinted genes in mice. More recent, with the advent of genome wide approaches, investigators have generated large datasets from F1 individuals generated from the breeding of two inbred (homozygous) strains of mice [28]. An advantage of using mice to do this type of work is that most strains have been sequenced and all animals within a strain will have the same maternal and paternal DNA sequence. While useful, the mouse model does not always faithfully represent other mammals [29]. In addition, most laboratory mice are inbred (homozygous) while other mammals are heterozygous which incorporates complexity to the analysis of identifying parental alleles. As imprinted gene expression is species-specific, tissue-specific, and developmental stage specific [24], investigators would have to do monetary and animal expensive studies to identify novel imprinted genes and their potential function in health and disease.

A current limitation for investigators working in the area of genomic imprinting in heterozygote animals such as bovine, is the difficulty to assess whether a gene or a region in a gene has ASE for the entire genome. For example, in the case in which 4 fetuses are obtained from the breeding of one cow and one bull, each of the fetuses may have a specific combination of alleles (penitentially 4 combinations), making the identification of imprinted gene expression a daunting task, not to mention extremely expensive. Therefore, new computational tools and analyses must be devised in order to provide investigators knowledge of allelic imbalances in the transcriptome which may then be used to do locus-specific wet bench work to determine the accuracy of the predictions.

Specifically, [23] measured gene expressions of four normal female F1 conceptuses (fetus and placenta) generated from the mating of Bos taurus (mother) and Bos taurus indicus (father). Tissues were retrieved from the brain, kidney, liver, skeletal muscle, and placenta of these four conceptuses. RNA-seq experiments were conducted on each tissue type for each replicate.

Aligning RNA-seq reads to a non-identical reference genome has been shown to introduce alignment bias [30, 31]. To address the mapping bias problem, [23] combined the reference genome (i.e., the *B. t. taurus* reference genome UMD3.1 build) and the pseudo *B. t. indicus* genome to create a custom diploid genome. Specifically, the sire’s DNA was subjected to next generation sequencing (DNA-seq) to identify all SNPs between his genome and the *B. t. taurus* reference genome. Then Genome Analysis Toolkit (GATK) [32] and SAMtools [33] pipelines were applied for SNP calling and only SNPs identified by both pipelines were used to generate a pseudo *B. t. indicus* genome. At last, RNA-seq reads from the *B. t. indicus* ×*B. t. taurus* F1 conceptuses were mapped to the diploid genome using both the HISAT2 [34] and BWA [35] pipelines and only variants identified by both methods were retained to minimize the potential effects of false positives. The resulting datasets are publicly available at the Gene Expression Omnibus database under accession number GSE63509.

We used the BLMRM method to separately analyze liver, kidney, muscle, and brain tissue data from [23]. Missing values are not uncommon in real datasets, especially when dealing with heterozygous species (for example, cattle and humans), as not all replicates share the same set of SNPs among parental alleles. We first filtered out genes containing only one SNP or for which all SNPs were not represented by at least two individuals. We also removed genes for which the observed maternal and paternal expression percentages were constant across all replicates and all SNPs as statistical inferences are straightforward in such a scenario. In total, 9,748 genes remained for analysis, among which many had low numbers of total RNA-seq read counts.

We then applied the proposed BLMRM method to these 9,748 genes. Hyperparameters were estimated using the method described in the “Method” section. For example, for liver tissue, we have $\widehat {\mu }=0.43$, $\widehat {\sigma }^{2}$= 4.62, $\widehat {a}_{s}=2.35$, $\widehat {b}_{s}=1.37$, $\widehat {a}_{r}=2.03$, and $\widehat {b}_{r}=0.09$.

**Fig. 1:**
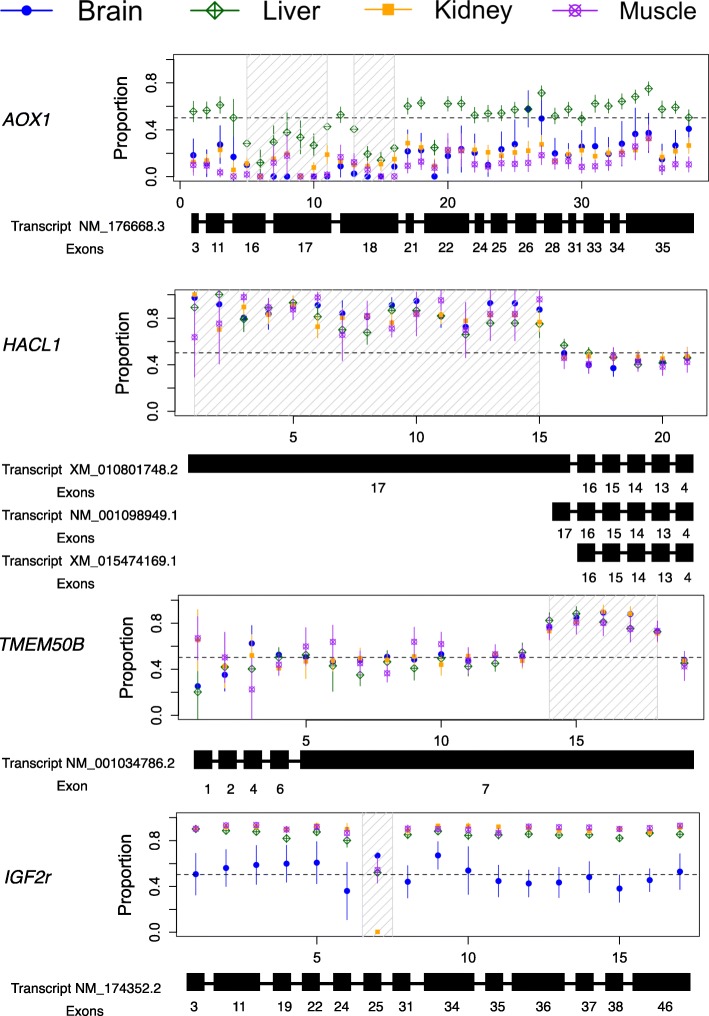
Percentage of gene expression from maternal allele in brain, liver, kidney, and muscle, respectively. The top panel shows gene *AOX1*. The second panel shows gene *HACL1*. The third panel shows gene *TMEM50B*, and the bottom panel shows gene *IGF2r*. SNPs are drawn with ascending genomic locations. The bottom of each panel shows distribution of SNPs in exons from all RefSeq annotated transcripts of this gene. Rectangles represent exons (only those with SNPs are shown) with exon numbers indicated under each rectangle. Lengths of exons are not drawn to scale

We identified several examples containing varied and informative patterns of tissue-specific and/or exon-specific ASEs. Here, we present four genes: *AOX1*, *HACL1*, *TMEM50B*, and *IGF2R*. Aldehyde oxidase 1 (*AOX1*; XLOC_003018) is a cytosolic enzyme expressed at high levels in the liver, lung, and spleen but at a much lower level in many other organs since this gene plays a key role in metabolizing drugs containing aromatic azaheterocyclic substituents [36, 37]. By controlling FDR at 0.05, the BLMRM method identified gene *AOX1* as exhibiting ASE at the gene level in the brain, kidney, and muscle, and biallelically expressed in the liver (top panel in Fig. 1). The vertical axis in Fig. 1 indicates the observed sample average percentage of gene expression from the maternal allele. The bar around each sample average denotes the 95% confidence interval at each SNP. SNPs are drawn with ascending genomic locations in a chromosome. The bottom of each panel in Fig. 1 shows the distribution of SNPs in exons from annotated RefSeq transcripts of this gene. Conclusions from our BLMRM method coincide with *AOX1* gene functional analysis. Using the binomial exact test, [23] only found that *AOX1* had preferential paternal expression in bovine muscle and failed to detect ASE in the brain and kidney. Our proposed method also suggests significant ASE variations across SNPs in the liver, kidney, and muscle with FDR at the 0.05 level. Interestingly, regions in the liver showing ASE variations corresponded to the 16th, 17th, and 18th exons housing the 5-7th and 14-16th SNPs. Given this exon- and tissue-specific information, biologists can examine the ASE regulatory mechanism in detail.

2-hydroxyacyl-CoA lyase (*HACL1*; XLOC_001524) is involved in perixosomal branched fatty acids oxidation and primarily expressed in the liver [38]. Our proposed method identified *HACL1* as exhibiting significant ASE at the gene level and its variations across SNPs. Figure 1 Panel 2 visualizes our observations and shows a clear maternal preference of expression for the first 15 SNPs, whereas the remaining six suggest biallelic expression of this gene. This surprising finding spurred further investigation, upon which we identified that the first 15 SNPs belong to exon 17 of alternative splice variant XM_010801748.2 while the last SNPs are shared between two or three splice isoforms (i.e. NM_001098949.1, XM_015474169.1, and XM_010801748.2). No further information is available regarding the ASE mechanism of this gene, as this is the first time we have retrieved such detailed statistical results for each gene in an entire genome within a short computational window. Future work will identify whether this ASE gene is a novel imprinted gene and if, in fact, this gene shows variant-specific imprinted expression as has been documented for other genes [39].

Transmembrane protein 50B (*TMEM50B*; XLOC_000329) is a ubiquitously expressed housekeeping gene. Our method identified this gene to be biallelically expressed in all analyzed tissues (Fig. 1, Panel 3) as expected for a housekeeping gene. Interestingly, our proposed method also predicted significant variations across SNPs in each of these four tissue types. Upon investigating detailed activity of this gene, Fig. 1 indicates that a portion of the 3’ UTR of this transcript appears to have maternal preference. The consistent pattern across tissues motivated us to understand the importance of this SNP variation. We hypothesize that this corresponds to a specific RNA variant required for maintaining cellular function.

Finally, insulin-like growth factor 2 receptor (*IGF2r*; XLOC_018398) is a well-known maternally expressed mannose receptor that targets IGF2 for degradation [40]. This gene is imprinted in the liver, kidney, and muscle (Fig. 1, Panel 4) but has biallelic expression in the brain of mice and cattle [41, 42]. In addition, *IGF2r* is lowly expressed in the cattle brain [42]. Prediction results from our proposed method coincide with the literature.

**Fig. 2:**
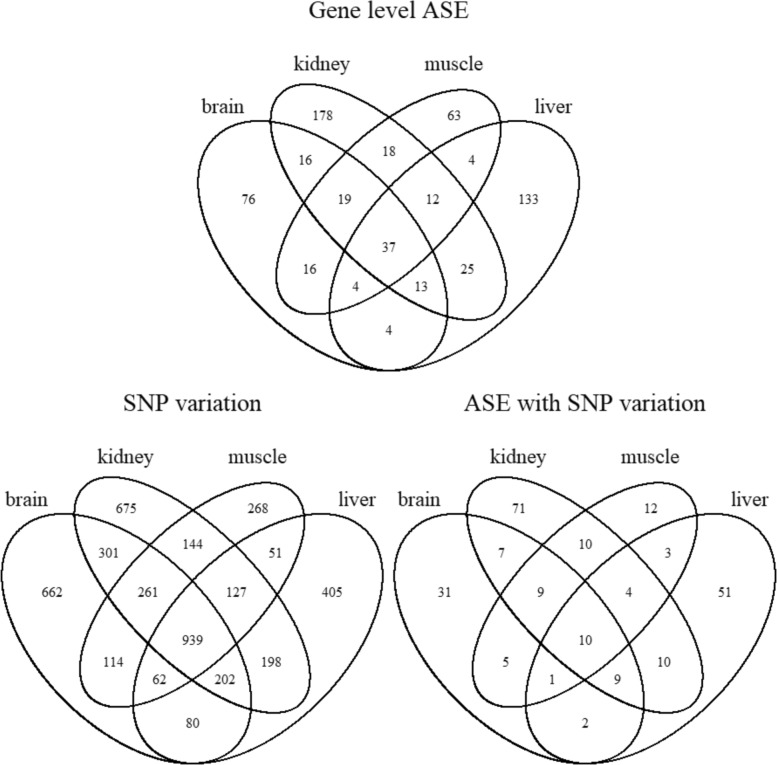
Venn Diagram of detected ASEs across tissue types. Number of significant genes (estimated FDR=0.05) across four tissue types when testing ASE at the gene level, testing ASE variations across SNPs, and testing ASE gene and ASE variations within a gene simultaneously

By controlling FDR at 0.05, Fig. 2 summarizes the numbers of detected ASE genes, numbers of genes with ASE variations across SNPs, and numbers of genes exhibiting ASE at the gene level and ASE variations across SNPs simultaneously, respectively, among the four tissues. We conducted some further analysis on these detected genes. For instance, in the top Venn diagram, among the 37 detected ASE genes shared by all four tissue types, 11 of them cannot be mapped to the set of annotated genes using the UMD 3.1 build. Among the rest of 26 annotated and detected ASE genes, we found that three of them had been documented as imprinted genes across all or most of these four tissue types. These three imprinted genes are (1) *GSTK1* that is maternally expressed in human placenta but unknown in other human tissues [43], paternally expressed in mouse kidney, liver, muscle, and maternally expressed in mouse brain [44], maternally expressed in bovine oocyte and unknown in other bovine tissues [45]; (2) *PLAGL1* that is paternally expressed in human kidney, muscle, and unknown in other human tissues [46], paternally expressed in mouse muscle, kidney, and brain [44], and paternally expressed in bovine brain, kidney, muscle, and liver [47]; (3) *BEGAIN*, which is unknown in human genome, preferentially expressed from the paternal allele in mouse neonatal brain [48], paternally expressed in bovine kidney and muscle with strong statistical evidence though no biological verification yet [42], and found to be paternally expressed in sheep kidney, liver, muscle, and brain (all four) tissue types [49]. Excluding these three documented imprinted genes, the other 23 annotated ASE genes detected by our BLMRM method are *de novo* detected ASE genes and their biological relevance await experimental verification.

Collecting all ASE genes from the first Venn diagram in Fig. 2, we summarized the number of detected ASE genes on each chromosome (see Additional file 1: Table S1). We found several interesting patterns. For instance, chromosomes 11 and 21 tend to have more ASE genes than other chromosomes for all tissue types. Besides, the X chromosome has more ASE genes in brain tissue than other tissue types. Additional file 1: Figure S1 plots distributions of these ASE genes in each chromosome, revealing several ASE clusters. Among all detected ASE genes, most ASE genes show preference of the maternal allele than the paternal allele. Specifically, 79%, 74%, 68%, and 71% ASE genes show maternal preference in the brain, liver, kidney, and muscle tissues, respectively.

At this stage, we are not able to statistically distinguish imprinted genes from other type of ASE genes as further experiment data are required to separate imprinting from other ASE molecular mechanisms. However, collecting all the detected ASE genes from all three Venn diagrams in Fig. 2, we found that seven *de novo* detected ASE genes are highly likely to be imprinted in the bovine genome but they have not been documented in any bovine study. They are: (1) *GATM*, *SNX14*, and *NT5E*, which are imprinted in mouse [50, 51]; (2) *IGF1R* and *RCL1*, which are imprinted in human [52, 53]; and (3) *KLHDC10* and *SLC22A18*, which are imprinted in both human and mouse [54, 55]. These genes are involved in varied physiological functions. For example, *GATM* encodes an arginine glycine amidinotransferase (AGAT) which is involved in creatine synthesis [56, 57]. *NT5E* encodes the protein CD73 (cluster of differentiation 73), a cell surface anchored molecule with ectoenzymatic activity that catalyzes the hydrolysis of AMP into adenosine and phosphate and has been shown to mediate the invasive and metastatic properties of cancers [58, 59]. *SNX14* is a protein coding gene involved in maintaining normal neuronal excitability and synaptic transmission [51] and may be involved in intracellular trafficking [60]. *IGF1R* is a receptor tyrosine kinase that mediates the actions of insulin-like growth factor 1 (IGF1). *IGF1R* is involved in cell growth and survival and has a crucial role in tumor transformation and survival of malignant cells [61, 62]. *RCL1* is a protein-coding gene with roles in 18 S rRNA biogenesis and in the assembly of the 40 S ribosomal subunit [63, 64]. The Kelch repeat protein *KLHDC10* activates the apoptosis signal-regulating kinase 1 (ASK1) through the suppression of protein phophatase 5 [65] and activation of the ASK1 contributes in oxidative stress-mediated cell death through the activation of the JNK and p38 MAPK pathways [66]. *SLC22A18* plays a role in lipid metabolism [67] and also acts as a tumor suppressor [68]. Visualization of significant expression pattern of these seven genes are plotted in Additional file 1: Figure S2 along with its significance level assessed by FDR.

### Study on simulated data

#### Simulation design

Simulation studies based on real datasets can best evaluate empirical usage and performance. In this subsection, we introduce our approach to simulate data based on the real dataset in [23]. In the next subsection, we will compare the BLMRM method with the binomial test, ANOVA, MBASED, generalized linear mixed model (GLMM), and the BLMRM method with pure Laplace approximation.

In each simulation, we simulated 4000 genes in total with 1000 genes for each of the four models in $\mathcal {M}$. To base our simulation upon real datasets, we randomly selected 4000 genes from liver tissue in the real dataset and used the numbers of SNPs of these genes as the numbers of SNPs for the 4000 simulated genes. To ensure consistency with the real dataset, we set the number of biological replicates to be four.

Real data from liver tissue in [23] indicates a linear relationship between the logarithm of average total read counts and that of the sample standard deviation of total read counts within a gene across SNPs. Real data also indicates a roughly linear relationship between the logarithm of average total read counts and that of the sample standard deviation of total read counts within a SNP across four replicates. To simulate *n*_*gjk*_, we utilized these two linear relationships. Specifically, let $\bar {n}_{g}$ denote the sample average of the total read count of gene *g* across SNPs; that is, $\bar {n}_{g}=\sum ^{J_{g}}_{j=1}(\bar {n}_{gj})/J_{g}$ where $\bar {n}_{gj}=\sum _{k=1}^{K} n_{gjk}$/K. For the liver tissue in real data, by regressing $\text {log} S(\bar {n}_{g})$ on $\text {log} (\bar {n}_{g})$ with a simple linear model where *S*(·) denotes the sample standard deviation, we obtained fitted intercept $\widehat {\alpha }_{1}=-0.36$ and slope $\widehat {\alpha }_{2}=0.97$. Hence, for each simulated gene, we independently sampled $\text {log }\bar {n}_{g1}, \dots, \text {log }\bar {n}_{{gJ}_{g}}\sim \text {N }(\mu =\text {log }\bar {n}_{g}$, and $\sigma =\widehat {\alpha }_{1}+\widehat {\alpha }_{2}\text {log }\bar {n}_{g})$, where $\bar {n}_{g}$’s were computed from the 4,000 genes randomly selected from the real dataset. Next, we fit a linear regression model between $\text {log} S(\bar {n}_{gj})$ and $\text {log} (\bar {n}_{gj})$, which yielded an estimated intercept $\widehat {\alpha }_{3}=-0.53$ and slope $\widehat {\alpha }_{4}=0.77$. Similarly, we simulated $n_{gj1}, \dots, n_{gj4}\sim \text {N }(\mu =\text {log }\bar {n}_{gj}, \sigma =\widehat {\alpha }_{3}+\widehat {\alpha }_{4}\text {log }\bar {n}_{gj})$. We rounded the simulated values to ensure *n*_*gjk*_’s were integers.

Given the simulated *n*_*gjk*_’s, to simulate *y*_*gjk*_’s, we needed to simulate *p*_*gjk*_’s. We simulated gene effect *β*_*g*_ uniformly from {−4.39,−1.20,−0.41,0.41,1.20,4.39} for genes where *β*_*g*_≠0. 0.41, 1.20, and 4.39 are the 10th, 50th, and 90th percentiles of absolute values of $\widehat {\beta }_{g}$’s, respectively, when significant gene ASEs are reported by the GLMM in (1). We simulated $\sigma ^{2}_{sg}\stackrel {iid}{\sim } \text {IG }(\widehat {a}_{s}, \widehat {b}_{s})$, $S_{gj}\stackrel {iid}{\sim }\text {N }(0, \sigma ^{2}_{sg})$, and simulated $\sigma ^{2}_{rg}\stackrel {iid}{\sim } \text {IG }(\widehat {a}_{r}, \widehat {b}_{r})$, $R_{gk}\stackrel {iid}{\sim }\text {N }(0, \sigma ^{2}_{rg})$, where $\widehat {a}_{s}$, $\widehat {b}_{s}$, $\widehat {a}_{r}$, and $\widehat {b}_{r}$ are hyperparameter estimates from the liver tissue whose values are given in real data analysis section. *p*_*gjk*_ was computed as exp(*β*_*g*_+*S*_*gj*_+*R*_*gk*_)/(1+exp(*β*_*g*_+*S*_*gj*_+*R*_*gk*_)). At last, we simulated *y*_*gjk*_∼Binomial(*n*_*gjk*_,*p*_*gjk*_). We repeated such simulation 10 times to assess variations in performance.

#### Simulation results

We compared our BLMRM method with the binomial test, ANOVA test in [12], MBASED method in [14], and GLMM in (1) without Bayesian priors. The binomial test and ANOVA test only detect the gene effect; the MBASED method can detect gene ASE and SNP variation separately but not simultaneously; and the GLMM and BLMRM methods can detect the gene effect, SNP variation, and gene ASE and SNP variation simultaneously. For the binomial, ANOVA, MBASED, and GLMM methods, we applied Storey’s method [69] to estimate and control FDR. The FDR control for our BLMRM method was described in the “Method” section.

For the proposed BLMRM method, the hyperparameter estimation is accurate and stable across 10 simulations. The mean of absolute biases across 10 simulations are 0.61, 0.12, 0.08, and 0.06, respectively, for $\widehat {a}_{s}$, $\widehat {b}_{s}$, $\widehat {a}_{r}$, and $\widehat {b}_{r}$; and the standard deviations of these 10 absolute biases are 0.17, 0.08, 0.04, and 0.00.

Table 1 summarizes the average true FDR and average true positive rate (TPr) across 10 simulations when we control the estimated FDR at 0.05. Numbers in parentheses are sample standard deviations. Results suggested that among all methods under investigation, only our proposed method controlled FDR at the nominal level. The BLMRM method with pure Laplace approximation did not control FDR for simultaneous test on both gene effect and SNP variation. In addition, the proposed BLMRM method also had slightly higher TPr than the pure Laplace approximation approach in testing SNP variation. This suggested that the combined method of empirical Bayes and Laplace approximation provided more accurate results than three layers of Laplace approximation. The GLMM method was slightly liberal in testing gene ASE, overly conservative in testing the random SNP effect, and overly liberal in testing simultaneous gene ASE and SNP variation. The MBASED and binomial test methods did not control FDR when testing the gene effect. The MBASED method can not test gene ASE and ASE variation across SNPs simultaneously. Thus, under our simulation scenario, the MBASED method did not correctly separate observed variations among multiple sources of variations; i.e., gene ASE, SNP variation, biological variation, and error variation.

**Table 1:** Assess of FDR control and TPr when controlling estimated FDR at 0.05

Method	True FDR			TPr(%)		
	gene	SNP	gene-SNP	gene	SNP	gene-SNP
BLMRM	0.053	0.028	0.059	66.37	60.82	17.51
	(0.006)	(0.004)	(0.014)	(0.87)	(1.80)	(1.65)
BLMRM	0.060	0.030	0.094	68.87	56.82	17.50
(pure Laplace)	(0.006)	(0.002)	(0.008)	(0.29)	(1.19)	(0.91)
GLMM	0.073	0.006	0.625	68.66	57.20	86.72
	(0.010)	(0.002)	(0.004)	(1.52)	(1.49)	(0.86)
MBASED	0.358	0.032	-	91.34	64.32	-
	(0.006)	(0.005)	-	(0.54)	(1.51)	-
ANOVA	0.194	-	-	82.02	-	-
	(0.007)	-	-	(1.04)	-	-
Binomial	0.314	-	-	88.26	-	-
	(0.003)	-	-	(0.80)	-	-

**Fig. 3:**
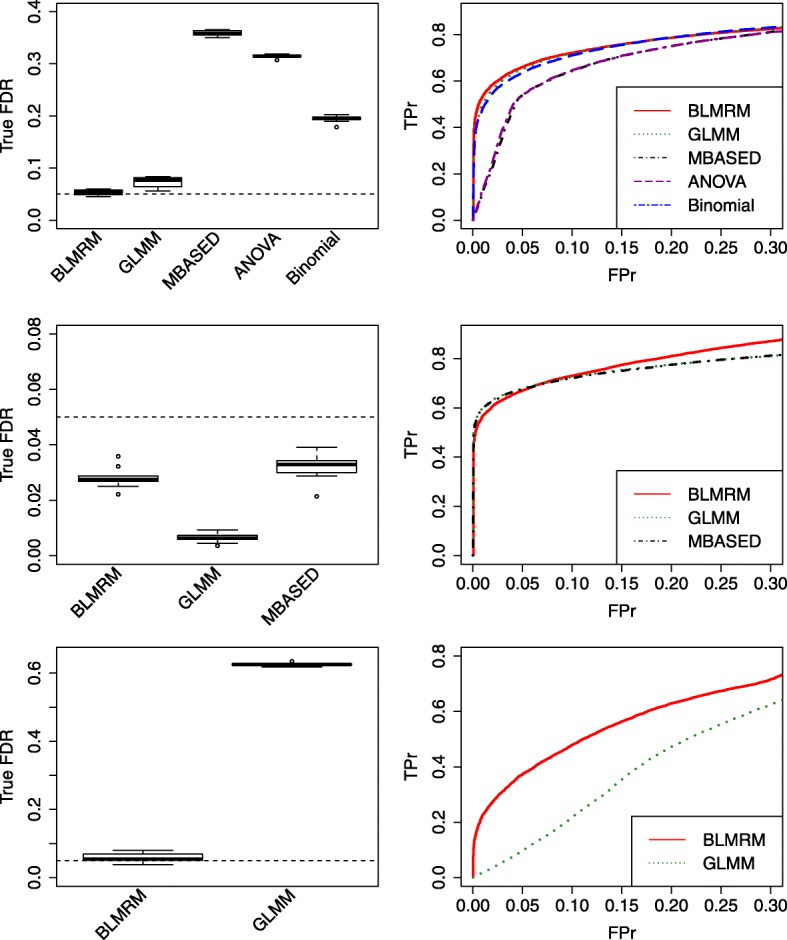
FDR and ROC comparison. Top row shows results for testing the gene effect; middle row shows results for testing SNP variation within a gene; bottom row shows results for simultaneously testing gene ASE and SNP variation. Left panel shows box plots of true FDR across 10 simulations when controlling estimated FDR = 0.05; right panel presents ROC curves

We plotted the box plots of true FDRs across 10 simulations in the left panel of Fig. 3, respectively, on testing the gene effect, SNP effect, and gene and SNP effects simultaneously when controlling the estimated FDR at 0.05, which represents same conclusions on FDR control in Table 1. The right panel in Fig. 3 displays the ROC curves when the false positive rate (FPr) was between 0 and 0.3. Compared to the other competing methods, the BLMRM method showed greater partial area under the ROC curves (AUCs) in testing gene ASE, SNP variation in ASE, and gene and SNP variation simultaneously. The GLMM and BLMRM methods were competitive for gene ranking when testing gene and SNP variation; however, the BLMRM method substantially outperformed the GLMM method in gene ranking when detecting simultaneous ASE gene effect and ASE variation within a gene.

## Discussion

So far, no existing statistical methods can provide simultaneous inferences at both gene and exon (SNPs) levels for the entire genome in a short computational window, like the *de novo* detection for the bovine genome shown here. We are able to achieve this goal because we model multiple sources of variations (i.e., genes, SNPs, biological replicates, error variation) in one statistical model and adopt an efficient estimation method (i.e., a combination of empirical Bayes and Laplace approximation) for model selection, that is designed for whole genome analysis.

## Conclusions

We have proposed a new method, BLMRM, to detect ASE for any RNA-seq experiment. Specifically, we propose a Bayesian logistic mixed regression model that accounts for variations from genes, SNPs, and biological replicates. To improve the reliability of inferences on ASE, we assign hyperpriors on genes, SNPs, and replicates, respectively. The hyperprior parameters are empirically estimated using observations from all genes in an entire genome. We then develop a Bayesian model selection method to test the ASE hypothesis on genes and variations of SNPs within a gene. To select a fitting model based on Bayes factors, we adopt a combination of the empirical Bayesian method and Laplace approximation method to substantially accelerate computation. To illustrate the utility of our method, we have applied the proposed approach to the bovine study that motivated our research; findings reveal the potential of our proposed method for application to real data analysis. We also conduct simulation studies that mimic the real data structure. Our data application and simulation study demonstrate the improved power, accuracy, and empirical utility of our proposed method compared to existing approaches. The R package, BLMRM, based on our method is available to download via Github at https://github.com/JingXieMIZZOU/BLMRM.

## Method

### Bayesian generalized linear mixed model

Let *n*_*gjk*_ denote the total number of read counts for the *k*th biological replicate of gene *g* at its *j*th SNP, where *g*=1,2,…,*G*,*j*=1,2,…,*J*_*g*_, and*k*=1,2,…,*K*. Let *y*_*gjk*_ denote the number of read counts from the maternal allele of replicate *k*. We model *y*_*gjk*_∼Binomial(*n*_*gjk*_,*p*_*gjk*_), where *p*_*gjk*_ denotes the proportion of gene expression from the maternal allele for gene *g* at SNP *j* of replicate *k*. It is known that using the RNA-seq approach to detect ASEs can produce bias during mapping because reads from the reference allele are more likely to be mapped due to fewer number of mismatches compared to reads from alternative alleles [30]. Potential solutions have been proposed in [23, 30, 70] to correct mapping bias. Here and throughout the paper, *n*_*gjk*_’s and *y*_*gjk*_’s denote the read counts after bias correction.

The objective of our study is to detect genes and regions within a gene whose expression is significantly different between the maternal and paternal alleles. Most existing methods assumed equal gene expression across all SNPs of a given gene; however, research discoveries have disproven this assumption for several reasons [71, 72], including alternative splicing and RNA variants. Thus, we model *y*_*gjk*_ as

1 $$\begin{array}{*{20}l} y_{gjk}\sim \text{Binomial}(n_{gjk},p_{gjk}), \text{ and}\\ \text{log}\frac{p_{gjk}}{1-p_{gjk}}=\beta_{g}+S_{gj}+R_{gk},  \end{array} $$

where *β*_*g*_ is the fixed gene effect; *S*_*gj*_ is the random SNP effect and $S_{gj}\stackrel {iid}{\sim }\mathrm {N}(0, \sigma ^{2}_{sg})$; *R*_*gk*_ is the random replicate effect and $R_{gk}\stackrel {iid}{\sim } \mathrm {N}(0,\sigma ^{2}_{rg})$. We also assume *S*_*gj*_’s and *R*_*gk*_’s are mutually independent. Therefore, the null hypothesis *H*_0_:*β*_*g*_=0 is to test whether gene *g* exhibits imbalanced allelic expression. Furthermore, $H_{0}: \sigma ^{2}_{sg}=0$ is to examine whether maternal (and/or paternal) gene expression percentage is the same across all SNPs of a gene.

Due to the expense of sample collection and sequencing experiments, most laboratories can only afford a few biological replicates, such as *K*= 3 or 4. In addition, the number of available SNPs in a gene also depends on the diversity between parental alleles. Often, only a small number of genes contain a large number of SNPs. Thus, for most genes, the estimates of *β*_*g*_, $\sigma ^{2}_{sg}$, and $\sigma ^{2}_{rg}$ are not robust, leading to unreliable statistical inferences. To improve estimation accuracy, we assume hierarchical priors on *β*_*g*_, $\sigma ^{2}_{sg}$, and $\sigma ^{2}_{rg}$ to share information across all genes in the genome. Specifically, we assume $\sigma ^{2}_{sg}\stackrel {iid}{\sim } \text {IG}(a_{s},b_{s})$, $\sigma ^{2}_{rg}\stackrel {iid}{\sim } \text {IG}(a_{r},b_{r})$, and a Gaussian prior on the gene effect *β*_*g*_∼*iid*N(*μ*,*σ*^2^). The hyperparameters *a*_*s*_, *b*_*s*_, *a*_*r*_, *b*_*r*_, *μ*, and *σ*^2^ no longer have the subscript *g* because they are estimated by pooling observations from all genes. Given that there are tens of thousands of genes in the genome, the estimates of these prior hyperparameters are accurate.

### Detection of imbalanced allelic gene expression through Bayesian model selection

Next, we describe our Bayesian model selection method to detect ASE at the gene level and corresponding variations across SNPs. Based on model (1), there are four models, indexed by *m*∈{1,2,3,4}, in model space $\mathcal {M}$, where *β*_*g*_=0 and $\sigma ^{2}_{sg}=0$ in Model 1; *β*_*g*_≠0 and $\sigma ^{2}_{sg}=0$ in Model 2; *β*_*g*_=0 and $\sigma ^{2}_{sg}\neq 0$ in Model 3; and *β*_*g*_≠0 and $\sigma ^{2}_{sg}\neq 0$ in Model 4. For each gene *g*, we select model *m* in $\mathcal {M}$, which has the largest posterior probability defined as

2 $$\begin{array}{*{20}l} P(m|\mathbf{y}^{g}, \mathbf{n}^{g}) &=\frac{P(m)P(\mathbf{y}^{g}|m, \mathbf{n}^{g})}{\sum_{m=1}^{4} P(m)P(\mathbf{y}^{g}|m, \mathbf{n}^{g})}\\ &\propto P(m)P(\mathbf{y}^{g}|m, \mathbf{n}^{g}),  \end{array} $$

where $\mathbf {y}^{g}=(y_{g11},\dots,y_{{gJ}_{g}K})'$ and $\mathbf {n}^{g}=(n_{g11},\dots,y_{{gJ}_{g}K})'$. *P*(*m*) denotes the prior probability of model *m*. Without prior information, we assume a uniform prior on space $\mathcal {M}$. Thus, our objective is to select a model *m* in $\mathcal {M}$ that maximizes the marginal likelihood *P*(**y**^*g*^|*m*,**n**^*g*^), which, when comparing two models, is equivalent to choosing the model *m* using the Bayes factor. Let **b**_*g*_ denote all random effects; that is, $\mathbf {b}_{g}=(S_{g1}, \dots, S_{{gJ}_{g}}, R_{g1}, \dots, R_{gK})'$. Hence,

3 $$\begin{array}{*{20}l} P(\mathbf{y}^{g}|m, \mathbf{n}^{g}) =\iiiint &P(\mathbf{y}^{g}|\beta_{g}, \mathbf{b}_{g}, \mathbf{n}^{g}, m) P(\beta_{g})\times\\ &P(\mathbf{b}_{g} | \sigma^{2}_{sg}, \sigma^{2}_{rg}) P(\sigma^{2}_{sg}, \sigma^{2}_{rg}) \times\\ &\,d \beta_{g} \,d {\mathbf{b}_{g}} \,d \sigma^{2}_{sg} \,d \sigma^{2}_{rg}.  \end{array} $$

A direct integration of (3) is difficult because an analytical result of the density is not a closed form. An alternative approach is to use Laplace approximation to iteratively approximate each integral; however, in our experience, this leads to error accumulated through each layer of integration and thus affects the accuracy of results. To overcome this problem, we propose a combination of empirical Bayes estimation and Laplace approximation. Inspired by the approach in [73], we obtain the following empirical Bayes estimators.

4 $$ \widetilde{\beta}_{g}=E(\beta_{g}|\widehat{\beta}_{g})\approx \frac{\widehat{\text{Var}(\beta_{g})}\widehat{\mu}+\widehat{\sigma}^{2}{\widehat{\beta}}_{g}}{\widehat{\text{Var}(\beta_{g})}+\widehat{\sigma}^{2}},   $$

5 $$ \widetilde{\sigma}_{sg}^{2}=E(\sigma_{sg}^{2}|\widehat{\sigma}_{sg}^{2})\approx\frac{d_{sg}\widehat{\sigma}_{sg}^{2}+2\widehat{b}_{s}}{d_{sg}+2\widehat{a}_{s}}, \text{ and}   $$

6 $$ \widetilde{\sigma}_{rg}^{2}=E(\sigma_{rg}^{2}|\widehat{\sigma}_{rg}^{2})\approx\frac{d_{rg}\widehat{\sigma}_{rg}^{2}+2\widehat{b}_{r}}{d_{rg}+2\widehat{a}_{r}},   $$

where $\widetilde {\beta }_{g}$, $\widetilde {\sigma }_{sg}^{2}$, and $\widetilde {\sigma }_{rg}^{2}$ denote the empirical Bayes estimates of *β*_*g*_, $\sigma ^{2}_{sg}$, and $\sigma ^{2}_{rg}$, respectively. $\widehat {\beta }_{g}$, $\widehat {\text {Var}(\beta _{g})}$, $\widehat {\sigma }_{sg}^{2}$, and $\widehat {\sigma }_{rg}^{2}$ are maximum likelihood estimates from model (1). $\widehat {\mu }$, $\widehat {\sigma }^{2}$, $\widehat {a}_{r}$, $\widehat {b}_{r}$, $\widehat {a}_{s}$, and $\widehat {b}_{s}$ are estimated hyperparameters whose estimation method will be introduced in detail later in this section. *d*_*rg*_ and *d*_*sg*_ are degrees of freedom of the random SNP and random replicate effect, respectively, with *d*_*sg*_=*J*_*g*_−1 and *d*_*rg*_=*K*−1. We enter these empirical Bayes estimates directly into (3), obtaining the approximation:

7 $$\begin{array}{*{20}l} P(\mathbf{y}^{g}|m, \mathbf{n}^{g})\approx \int &P(\mathbf{y}^{g}|\widetilde{\beta}_{g}, \mathbf{b}_{g}, m, \mathbf{n}^{g})\times\\ &P(\mathbf{b}_{g} | \widetilde{\sigma}^{2}_{sg}, \widetilde{\sigma}^{2}_{rg}) \,d {\mathbf{b}_{g}}.  \end{array} $$

Accordingly, (3) is reduced to (7), which requires only one step of Laplace approximation. Our objective in combining empirical Bayes estimates and Laplace approximation is to develop a method with improved power and accuracy while maintaining affordable computational power that allows for empirical application. In our simulation study, we compared our proposed approach with the method using pure Laplace approximation. We found that our proposed method is superior than purely using Laplace approximation with respect to FDR control and true positive rate (see “Simulation results” section). This approach also greatly decreases computational requirements compared to MCMC, considering there are tens of thousands of genes in an entire genome [74]. For instance, the method in [19] employs an MCMC algorithm for identifying ASE. With the default setting, their approach took approximately 1.5 hours to analyze 50 genes, whereas our method took approximately 3 minutes.

We still need to estimate hyperparameters *μ*, *σ*^2^, *a*_*s*_, *b*_*s*_, *a*_*r*_, and *b*_*r*_. To avoid extreme values that produce unstable estimates, we first let $y_{gjk}^{*} = y_{gjk} + 1$ and $n_{gjk}^{*} = n_{gjk} + 2$. Then, based on $y_{gjk}^{*}$’s and $n_{gjk}^{*}$’s, *μ* and *σ*^2^ are estimated by the method of moments using significant $\widehat {\beta }_{g}$ via likelihood ratio tests when controlling FDR at 0.05. *a*_*s*_, *b*_*s*_, *a*_*r*_, and *b*_*r*_ are estimated based on $y_{gjk}^{*}$’s and $n_{gjk}^{*}$’s by the maximum likelihood method, where *a*_*s*_ and *b*_*s*_ are based on significant estimates of $\widehat {\sigma }^{2}_{sg}$’s via likelihood ratio tests and controlling FDR at 0.05, and *a*_*s*_ and *b*_*s*_ are based on $\widehat {\sigma }^{2}_{rg}$’s from all genes.

Finally, we test *H*_0_:*β*_*g*_=0 and $H_{0}: \sigma ^{2}_{sg}=0$ for gene *g* by choosing Model *m*, where $m=\underset {\gamma \in \{1,2,3,4\}}{\arg \max } \text {} P(\gamma |\mathbf {y}^{g}, \mathbf {n}^{g})$ for *g*=1,…,*G*. Let *P*(*g*∈{*m*}|**y**^*g*^,**n**^*g*^) denote the posterior probability of gene *g* being sampled from Model *m*. The posterior probability of a gene exhibiting an ASE gene effect is *P*(*g*∈{2,4}|**y**^*g*^,**n**^*g*^). Similarly, the posterior probability of a gene exhibiting ASE variations across SNPs is *P*(*g*∈{3,4}|**y**^*g*^,**n**^*g*^). Finally, the posterior probability of a gene exhibiting an ASE gene effect and ASE variations across SNPs simultaneously is *P*(*g*∈{4}|**y**^*g*^,**n**^*g*^). We adopt the following method to control FDR that have been used in [74, 75]. To control the FDR when testing the ASE gene effect, we order *P*(*g*∈{2,4}|**y**^*g*^,**n**^*g*^), *g*=1,…,*G*, from largest to smallest. Let *g*_(1)_,…,*g*_(*G*)_ be the ordered genes; then, we find the largest *l* such that $\sum ^{l}_{i=1}(1-P(g_{(i)}\in \{2,4\}|\mathbf {y}^{g_{(i)}}, \mathbf {n}^{g_{(i)}}))/l \leq \alpha $, where *α* is a pre-defined FDR threshold. We declare the first *l* genes are significant for testing *H*_0_:*β*_*g*_=0 when FDR is controlled at *α* level. The same strategy is used to control FDR for testing ASE variations among SNPs and gene and SNP variation effects simultaneously.

## Supplementary information

**Additional file 1** Supplementary Materials for “Modeling Allele-Specific Expression at the Gene and SNP Levels Simultaneously by a Bayesian Logistic Mixed Regression Model”.

## Data Availability

The allele-specific expression data for the bovine study are publicly available at Gene Expression Omnibus with accession no. GSE63509. The R package, BLMRM, is publicly available at https://github.com/JingXieMIZZOU/BLMRM.

## Abbreviations

ANOVA: Analysis of variance
ASE: Allele-specific expression
AUC: Area under ROC curve
BLMRM: Bayesian logistic mixed regression model
BWS: Beckwith-Wiedemann syndrome
DNA-seq: next generation sequencing of DNA
FDR: False discovery rate
FPr: False positive rate
GATK: Genome Analysis Toolkit
GLMM: Generalized linear mixed model
MCMC: Markov chain Monte Carlo
SNP: Single nucleotide polymorphism
TPr: True positive rate
