# Supplementary material for: Modeling allele-specific expression at the gene and SNP levels simultaneously by a Bayesian logistic mixed regression model
Source: BMC Bioinformatics. 2019 Oct 28;20:530. doi: 10.1186/s12859-019-3141-6 (PMC6819473; doi:10.1186/s12859-019-3141-6)
Supplement: Supplementary file 1 — Additional file 1 Supplementary Materials for “Modeling Allele-Specific Expression at the Gene and SNP Levels Simultaneously by a Bayesian Logistic Mixed Regression Model”. [file 12859_2019_3141_MOESM1_ESM.pdf]

Supplementary Materials for "Modeling Allele-Specific Expression at the Gene and SNP Levels Simultaneously by a Bayesian Logistic Mixed Regression Model"

Jing Xie[1], **Tieming Ji**[1*], **Marco A. R. Ferreira**[2], **Yahan Li**[3], **Bhaumik N. Patel**[3], **Rocio M. Rivera**[3]

[1] Department of Statistics, University of Missouri at Columbia, Columbia, MO, USA, 65211

[2] Department of Statistics, Virginia Tech, Blacksburg, VA, USA 24601

[3] Division of Animal Science, University of Missouri at Columbia, Columbia, MO, USA, 65211

*email: jit@missouri.edu

1 Web Appendix A: Distribution of detected ASEs in the bovine genome across tissue types

Supplementary Table 1 summarizes the number of detected ASEs on each chromosome for each tissue type.

Supplementary Table 1: Distribution of ASE genes on each chromosome for four different tissues.

chromosome	Brain	Liver	Kidney	Muscle	chromosome	Brain	Liver	Kidney	Muscle
1	8	8	12	7	16	7	12	13	8
2	10	7	9	7	17	0	6	6	4
3	7	16	14	4	18	5	11	13	5
4	6	5	7	7	19	5	6	9	3
5	4	14	16	8	20	4	1	5	6
6	4	8	11	6	21	13	14	17	10
7	7	9	10	5	22	6	7	6	4
8	8	13	15	6	23	3	8	11	3
9	4	7	9	7	24	5	9	6	6
10	2	6	11	5	25	5	3	11	2
11	12	14	19	10	26	1	1	8	4
12	6	0	6	4	27	4	6	8	3
13	7	8	13	8	28	1	3	6	3
14	6	10	11	3	29	3	7	13	5
15	4	5	7	6	*X*	28	8	16	14

Supplementary Figure 1 explores the distribution of ASE genes in each chromosome across tissue types. Specifically, dots represent ASE genes; horizontal axis shows genomic locations in base pairs. The three labeled locations show the first, median, and last positions of SNPs across tissue types. Since the median and last positions are too close in chromosome 17, we only label the last SNP location.

2 Web Appendix B: Expression pattern of seven potential imprinted genes in cattle genome

The detected ASE expression pattern of gene GATM, SNX14, NT5E, IGF1R, RCL1, KLHDC10, and SLC22A18 are plotted in the Supplementary Figure 2. Significance level, assessed by FDR, is labeled below each subfigure. Exons corresponding to SNPs are plotted under each subfigure also. During SNP analysis, we identified variants which are not found in exons of genes in the UMD3.1 build. However,

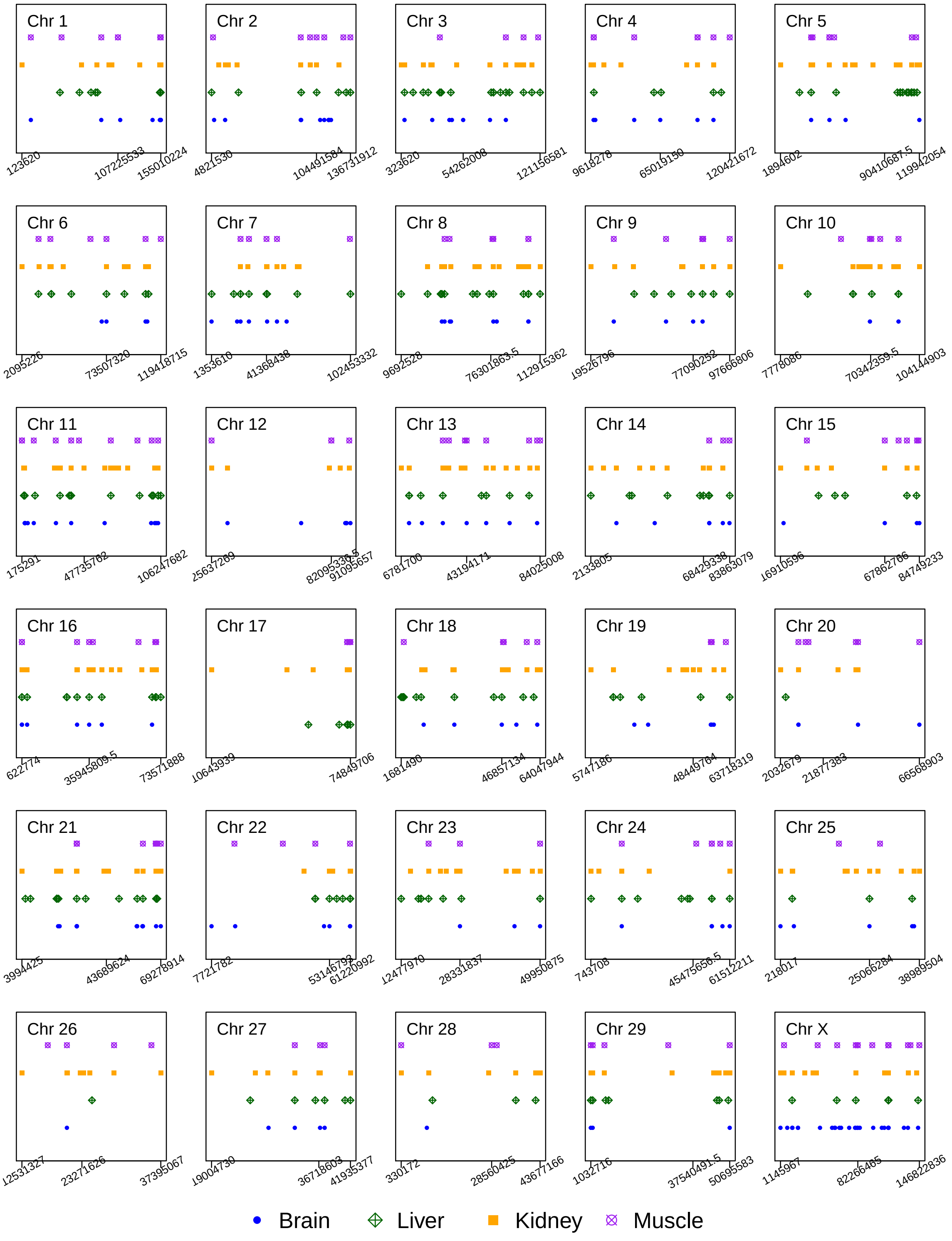

Supplementary Figure 1: ASE distribution on each chromosome for four different tissue types.

upon further investigation of these regions with the software Apollo and the Bovine Genome Database, alternative 3' UTR's or exons are supported for these regions by Iso-Seq reads in multiple tissues.

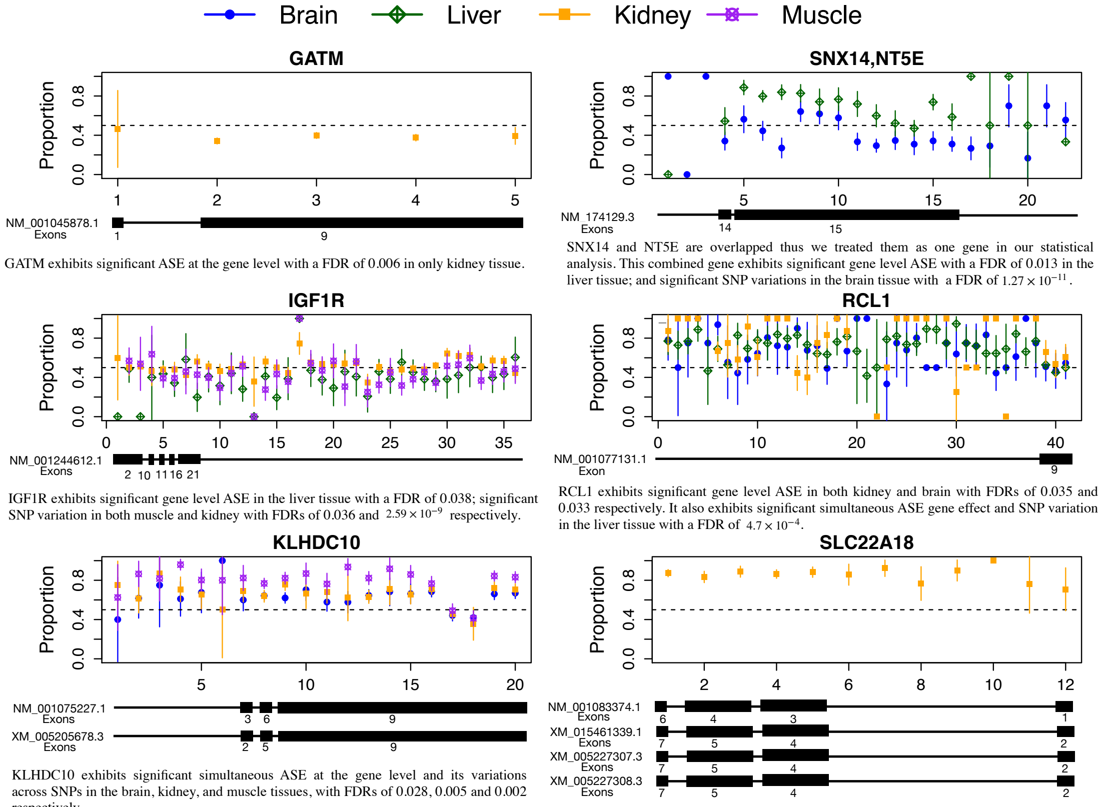

GATM exhibits significant ASE at the gene level with a FDR of 0.006 in only kidney tissue.

SNX14 and NT5E are overlapped thus we treated them as one gene in our statistical analysis. This combined gene exhibits significant gene level ASE with a FDR of 0.013 in the liver tissue; and significant SNP variations in the brain tissue with a FDR of 1.27×10^{-11}.

IGF1R exhibits significant gene level ASE in the liver tissue with a FDR of 0.038; significant SNP variation in both muscle and kidney with FDRs of 0.036 and 2.59×10^{-9} respectively.

RCL1 exhibits significant gene level ASE in both kidney and brain with FDRs of 0.035 and 0.033 respectively. It also exhibits significant simultaneous ASE gene effect and SNP variation in the liver tissue with a FDR of 4.7×10^{-4}.

KLHDC10 exhibits significant simultaneous ASE at the gene level and its variations across SNPs in the brain, kidney, and muscle tissues, with FDRs of 0.028, 0.005 and 0.002 respectively.

SLC22A18 exhibits significant ASE at the gene level with a FDR of 0.005 in only kidney tissue.

Supplementary Figure 2: Visualizations and statistical analysis results of the seven potential imprinted genes.
